# CD21^low^ B cells reveal a unique glycosylation pattern with hypersialylation and hyperfucosylation

**DOI:** 10.3389/fimmu.2025.1512279

**Published:** 2025-02-12

**Authors:** Peter Tobias Felixberger, Geoffroy Andrieux, Andrea Maul-Pavicic, Sigune Goldacker, Ina Harder, Sylvia Gutenberger, Jonathan J. M. Landry, Vladimir Benes, Till Fabian Jakob, Melanie Boerries, Lars Nitschke, Reinhard Edmund Voll, Klaus Warnatz, Baerbel Keller

**Affiliations:** ^1^ Department of Rheumatology and Clinical Immunology, Medical Center - University of Freiburg, Faculty of Medicine, University of Freiburg, Freiburg, Germany; ^2^ Center for Chronic Immunodeficiency (CCI), Medical Center - University of Freiburg, Faculty of Medicine, University of Freiburg, Freiburg, Germany; ^3^ Institute of Medical Bioinformatics and Systems Medicine, Medical Center - University of Freiburg, Faculty of Medicine, University of Freiburg, Freiburg, Germany; ^4^ Genomics Core Facility, European Molecular Biology Laboratory, Heidelberg, Germany; ^5^ Department of Oto-Rhino-Laryngology, Medical Center - University of Freiburg, Faculty of Medicine, University of Freiburg, Freiburg, Germany; ^6^ German Cancer Consortium (DKTK), Partner site Freiburg, a partnership between DKFZ and Medical Center - University of Freiburg, Freiburg, Germany; ^7^ Division of Genetics, Department of Biology, University of Erlangen, Erlangen, Germany

**Keywords:** CD21low B cells, CVID, glycome, glycosylation, hypersialylation, hyperfucosylation, anti-IgM/IFNγ, T-bet

## Abstract

**Background:**

The posttranslational modification of cellular macromolecules by glycosylation is considered to contribute to disease pathogenesis in autoimmune and inflammatory conditions. In a subgroup of patients with common variable immunodeficiency (CVID), the occurrence of such complications is associated with an expansion of naïve-like CD21^low^ B cells during a chronic type 1 immune activation. The glycosylation pattern of B cells in CVID patients has not been addressed to date.

**Objective:**

The objective of this study was to examine the surface glycome of B cells in patients with CVID and associated immune dysregulation.

**Methods:**

We performed surface lectin staining on B cells from peripheral blood and tonsils, both *ex vivo* and after *in vitro* stimulation. Additionally, we examined the expression of glycosylation-related genes by RNAseq in naïve-like CD21^low^ B cells *ex vivo*, as well as in naïve CD21^pos^ B cells from healthy controls after *in vitro* stimulation.

**Results:**

Unlike CD21^pos^ B cells, naïve-like CD21^low^ B cells from CVID patients and CD21^low^ B cells from healthy controls exhibited a unique glycosylation pattern with high levels of α2,6 sialic acids and fucose. This hypersialylation and hyperfucosylation were particularly induced by activation with anti-IgM and interferon-γ (IFN-γ). Transcriptome analysis suggested that naïve-like CD21^low^ B cells possess a comprehensively reorganised glycosylation machinery, with anti-IgM/IFN-γ having the potential to initiate these changes *in vitro*.

**Conclusion:**

CD21^low^ B cells are hypersialylated and hyperfucosylated. This may implicate altered lectin-ligand interactions on the cell surface potentially affecting the CD21^low^ B-cell function. These glycome changes appear to be driven by the prominent type I immune response in complicated CVID patients. A better understanding of how altered glycosylation influences immune cell function could lead to new therapeutic strategies.

## 1 Introduction

Common variable immunodeficiency (CVID) is the most prevalent human antibody deficiency syndrome. In addition to recurrent infections, up to 60% of CVID patients present with noninfectious complications, such as autoimmunity, lymphoproliferation, and organ inflammation (CVIDc) (1). B-cell differentiation in these patients is typically characterised by a reduction in class-switched memory (cs mem) B cells and plasma cells. A subgroup of these patients further presents with an accumulation of naïve-like CD21^low^ B cells, classified as CVID smB-21lo patients according to the EUROclass classification (2). CD21^low^ B cells constitute a B-cell subset that is characterised by the common and uniquely high expression of the transcription factor T-box expressed in T cells (T-bet) (3), most prominently induced by interferon-γ (IFN-γ) and B-cell receptor (BCR) costimulation, the integrin CD11c, and a strongly divergent gene expression profile compared to conventional B-cell subsets (4, 5). CD21^low^ B cells are infrequent in healthy individuals and expand not only in the peripheral blood of CVIDc patients but also in distinct autoimmune and infectious disease conditions such as systemic lupus erythematosus (SLE) (6), rheumatoid arthritis (RA) (7), Sjögren’s syndrome (8), chronic HIV infection (9), malaria (10) and others (11, 12). As the differentially expressed genes (DEGs) of CD21^low^ B cells contained some enzymes involved in glycosylation, and, in addition, an altered glycome has been suggested to contribute to disease pathogenesis in autoimmune disorders (13–18), we set out to explore the glycome of naïve-like CD21^low^ B cells of CVIDc patients in more detail. Indeed, our investigations uncovered, for the first time, comprehensive aberrations in the glycome of CD21^low^ B cells in CVID smB-21lo patients and healthy individuals, which might, in part, be direct subjects to the transcriptional regulation of T-bet.

## 2 Materials and methods

### 2.1 Patient cohort and ethics statement

The current study included blood from 23 patients (14 women, nine mem; age: median 50 years ± 13 years) who fulfilled the diagnostic criteria for CVID (www.ESID.org). All patients were classified as CVID smB-21lo according to the EUROclass classification (2) and suffered from additional immune dysregulation, as listed in **Supplementary Table S1**. Control blood and tonsils were obtained from healthy donors (HD) without known immunodeficiency. All experiments were performed with ethical approval from local authorities (251/13 and 254/19) in accordance with the Declaration of Helsinki. Written informed consent was obtained from all patients, healthy individuals, or their parents. Tonsillectomies were performed for medical reasons.

### 2.2 Antibodies and lectins used in this study

The following antibodies and lectins were used: CD3 PE/Cy7 (UCHT1), CD4 BV421 (RPA-T4), sialyl-Lewis X (SLe^X^) AF488 (FH6), CD19 APC/Cy7 (HIB19), CD21 PE/Cy7 (Bu32), CD27 BV421 (M-T271), CD38 PerCP/Cy5.5 (HIT2), IgD BV785 (IA6-2), IgM BV605 (MHM-88) were obtained from BioLegend (San Diego, CA, USA). CD3 BUV395 (UCHT1), CD27 BV605 (L128), IgG AF700 (G18-145), and streptavidin APC (70312) for the detection of biotinylated lectins were obtained from BD Biosciences (Franklin Lakes, NJ, USA). CD19 PE/Cy7 (J3-119), and CD21 PE (BL13) were obtained from Beckman Coulter (Brea, CA, USA). IgD PE (polyclonal), IgD FITC (polyclonal), and IgM Cy5 (polyclonal) were obtained from SouthernBiotech (Birmingham, AL, USA). *Sambucus nigra* agglutinin (SNA) FITC (ZE0328), *Lens culinaris* agglutinin (LCA) FITC (ZD0104), *Aleuria aurantia* lectin (AAL) FITC (ZE0628), and *Erythrina cristagalli* lectin (ECL) biotinylated (ZB042) were obtained from Vector Laboratories (Newark, CA, USA). Lectin-binding properties and symbol nomenclature for graphical representations of glycans (19) are shown in **Supplementary Figure S1**. All glycans were created using the web software DrawGlycan (20).

### 2.3 Cell isolation

PBMCs were isolated from EDTA blood by Ficoll density gradient centrifugation following standard protocols. For *in vitro* cultures, naïve B cells were isolated using the naïve B-Cell Isolation Kit II (Miltenyi Biotec, Bergisch Gladbach, Rhineland, Germany) following the manufacturer’s instructions. To determine the purity of naïve B cells, cells were stained with IgD FITC, CD21 PE, CD38 PerCpCy5.5, CD3 PC7, IgM Cy5, CD19 APC-Cy7, CD4 BV421, and CD27 BV605. Purity was above 93%. Tonsil cells were obtained by mechanical dissociation. After isolation, cells were resuspended in IMDM (Life Technologies, Carlsbad, CA, USA) with 10% foetal calf serum (FCS; Biochrom, Cambridge, Cambridgeshire, United Kingdom).

### 2.4 Flow cytometric staining

Harvested cells were washed two to three times in phosphate-buffered saline (PBS; Lonza Basel, Switzerland) 1% bovine serum albumin (BSA; Sigma-Aldrich St. Louis and Burlington, MA, USA). Cell surface lectin staining was performed separately for each lectin (SNA FITC, LCA FITC, AAL FITC ECL biotinylated) for 15 min on ice. Cells were washed with PBS containing 1% BSA. For ECL, cells were subsequently stained with streptavidin APC for 15 min on ice. Antibody staining was then performed for 15 min at 4°C. PBMCs and tonsil cells were stained with IgD PE, CD38 PerCpCy5.5, CD21 PC7, CD19 APC-Cy7, and CD27 BV421 to determine the respective B-cell subsets. For flow cytometric analysis of naïve activated B cells, cells were stained with IgD PE and CD19 PC7, and DAPI was added to exclude dead cells prior to data acquisition. Data were acquired using an LSRFortessa (BD Biosciences) and analysed using FlowJo software (Treestar, Ashland, OR, USA).

### 2.5 *In vitro* culture assay

For B-cell activation, 50,000 isolated naïve B cells were stimulated with anti-IgM (5 µg/ml; SouthernBiotech, Birmingham, AL, USA), IFN-γ (50 ng/ml; BioLegend), CpG oligodeoxynucleotides (2.5 µg/ml, InvivoGen, San Diego, CA, USA), or cultivated without additional stimulus in IMDM supplemented with l-glutamine, l-glutathione, insulin, apo-transferrin (all Sigma-Aldrich), nonessential amino acids (Gibco, Waltham, MA, USA), and 10% FCS for 48 h as described before (5). Cells were harvested and processed for flow cytometry or RNAseq.

### 2.6 RNAseq

After stimulation, B cells were resuspended in RLT buffer (Qiagen, Shanghai, China) and frozen at − 80°C. RNA was isolated using the RNAeasy Micro Kit (Qiagen) according to the manufacturer’s instructions. Samples were processed and sequenced as described previously (5). Alignments and gene count tables were obtained for each sample using STAR (version 2.4.2a) on the GRCh38 genome and annotation (GRCh38.p7). Differential gene expression was analysed using the limma R package. An adjusted *p*-value (Benjamini and Hochberg) < 0.05 was considered significant. The original raw files are available in the Gene Expression Omnibus data repository of the National Center for Biotechnology Information (21) under the accession number GSE181739 for anti-IgM/IFN-γ *in vitro*-stimulated B cells or GSE148163 for naïve-like CD21^low^ B cells *ex vivo*.

### 2.7 Statistics

Statistical analyses were performed using GraphPad Prism 10 (GraphPad Software Inc., San Diego, CA, USA). Normal distribution was determined using D’Agostino and Pearson or Shapiro–Wilk test. Data were analysed using repeated measures one-way ANOVA with Tukey’s multiple comparisons test for normally distributed results and Friedman’s test with Dunn’s multiple comparisons test for nonnormally distributed results, as indicated. *p*-values of less than 0.05 were considered significant: ^*^ *p* < 0.05; ^**^ *p* < 0.01; ^***^ *p* < 0.001; and ^****^ *p* < 0.0001. Error bars in all figures define the means ± standard deviation (SD).

## 3 Results

### 3.1 Cell surface glycome of CD21^low^ B cells

**Figure 1:**
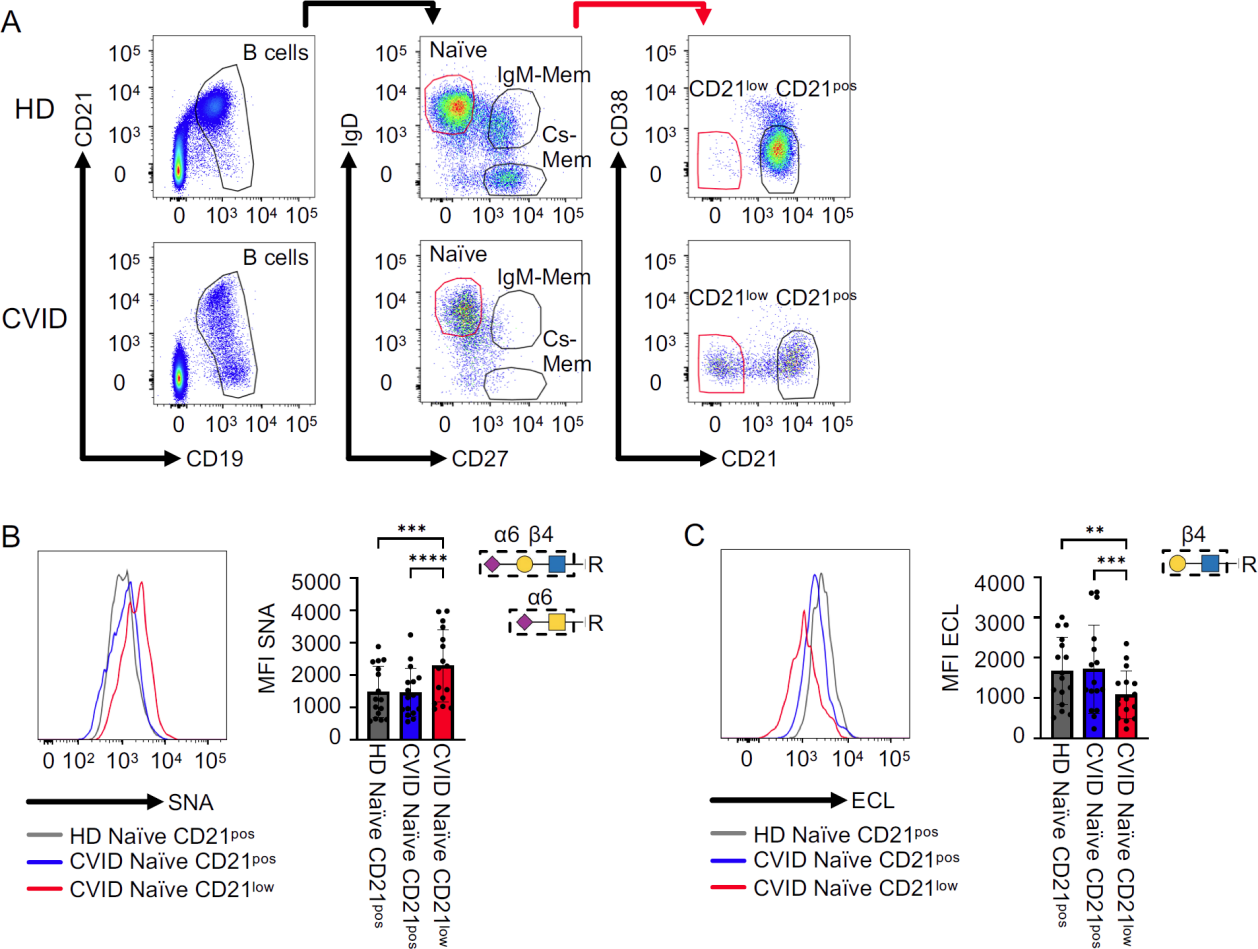
Sialylation on CVID patients’ B cells. **(A)** Naïve, IgM memory (IgM mem), and class-switched memory B cells (cs mem) were defined based on IgD and CD27 expression after gating on CD19^pos^CD21^pos-low^ B cells. Subpopulations were further differentiated into CD21^pos^ and CD21^low^ subsets. **(B)** Representative histogram for naïve B cells from one HD, naïve CD21^pos^, and naïve-like CD21^low^ B cells from a CVID patient, with corresponding statistical analysis of mean fluorescence intensities (MFI) after SNA staining (*n* = 19). **(C)** Representative histogram for ECL staining as described in **(A)** (*n* = 16). Lectin-binding epitopes are illustrated. *p*-values as determined by one-way ANOVA with Tukey’s multiple comparisons test **(B, C)**. **p < 0.01; ***p < 0.001; and ****p < 0.0001.

To address the question of whether the glycome of CVID patients’ B cells, and especially of CD21^low^ B cells, is altered, we performed lectin staining of B cells from CVID smB-21lo patients and compared these to their CD21^pos^ B-cell counterparts in patients and HD. Glycan binding properties for SNA (22, 23), ECL (22, 24), AAL (22, 25), LCA (22, 26, 27), and anti-SLe^X^ are shown in **Supplementary Figure S1**. The gating strategy for naïve, IgM memory (IgM mem), cs mem, and CD21^low^ B cells is depicted in **Figure 1A**. Due to the low memory cell numbers in CVID patients, we restricted the analysis of CD21^low^ B cells to the naïve B-cell compartment.

Lectin staining with α2,6 sialic acid-binding SNA (22, 23) revealed significantly increased levels of SNA ligands on the surface of naïve-like CD21^low^ B cells from CVID patients compared to naïve CD21^pos^ B cells from patients or HD. In contrast, patients’ naïve CD21^pos^ B cells were comparable to those from HD (**Figure 1B**). ECL binds terminal β1,4-linked galactose (22, 24), representing an asialylated form of SNA ligands (**Supplementary Figure S1**). In line with increased SNA binding, ECL staining demonstrated a reduction of ligands on the surface of naïve-like CD21^low^ B cells from patients compared to naïve CD21^pos^ B cells from patients and HD. Naïve CD21^pos^ B cells from patients and HD exposed ECL ligands to a similar extent (**Figure 1C**). Overall, these data reveal α2,6 hypersialylation of naïve-like CD21^low^ B cells from CVID patients.

**Figure 2:**
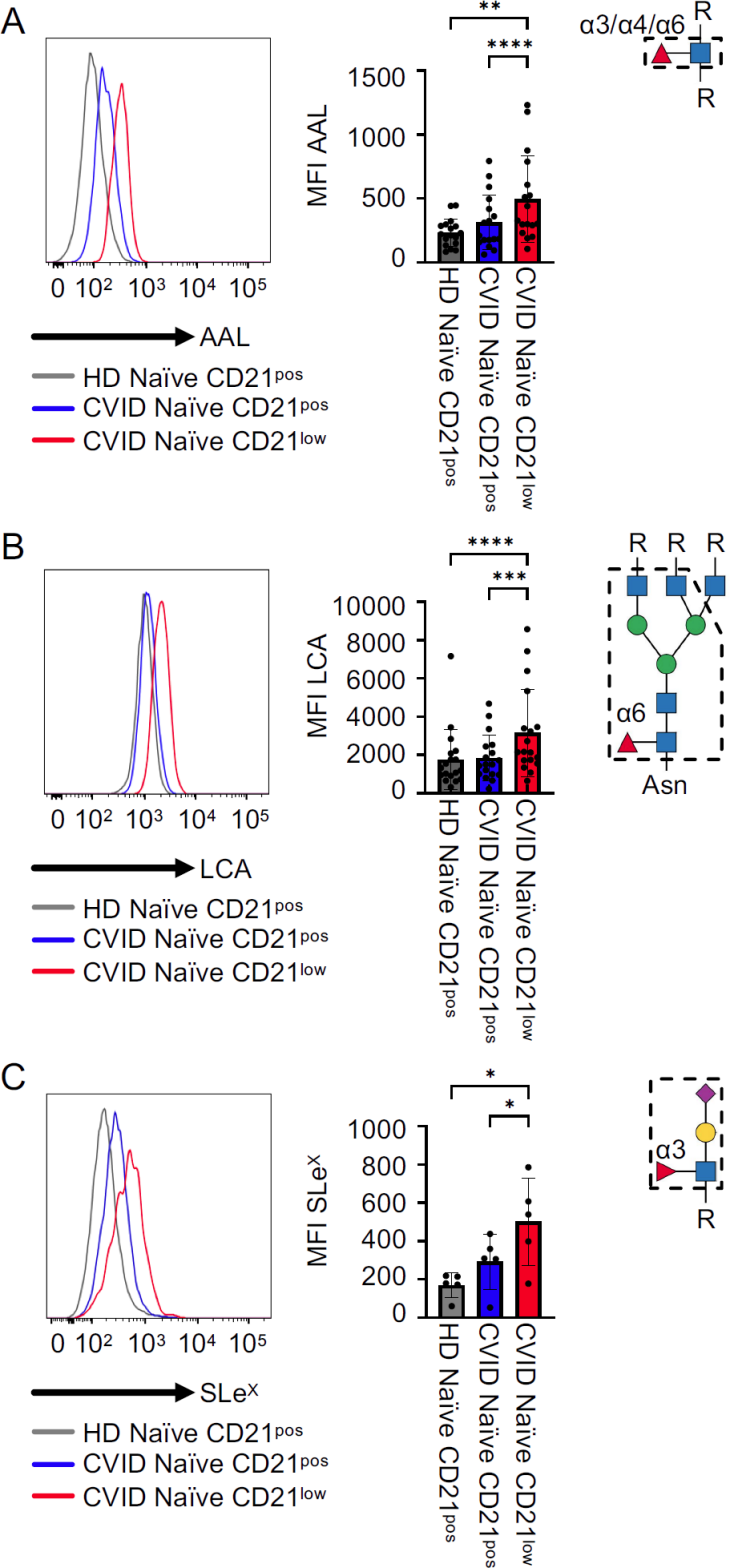
Fucosylation on CVID patients’ B cells. **(A)** Representative histogram for AAL in naïve CD21^pos^ B cells from a HD and naïve CD21^pos^ and naïve-like CD21^low^ B cells from a CVID patient, with corresponding statistical analysis (*n* = 20). **(B, C)** LCA (*n* = 18) and anti-SLe^X^ (*n* = 5) staining as described in **(A)**. Lectin/antibody-binding epitopes are illustrated. *p*-values as determined by one-way ANOVA with Tukey’s multiple comparisons test **(A, B)** and Friedman’s test with Dunn’s multiple comparisons test **(B)**. *p < 0.05; **p < 0.01; ***p < 0.001; and ****p < 0.0001.

AAL binds to α1,3-, α1,4-, and α1,6-fucosylated glycans (22, 25). Lectin staining revealed an increased expression of AAL ligands on the surface of naïve-like CD21^low^ B cells from CVID patients compared to naïve CD21^pos^ B cells from patients or HD (**Figure 2A**). Similarly, lectin staining with LCA, which binds selectively to N-glycans with α1,6-linked core fucose (22, 26, 27), was increased on naïve-like CD21^low^ B cells from CVID patients compared to naïve CD21^pos^ B cells from patients and HD (**Figure 2B**). The mean fluorescence intensity (MFI) of both lectins was similar in naïve CD21^pos^ B cells from patients and HD. Given the prominent role of SLe^X^, an α1,3 fucose-containing selectin ligand, on leukocyte trafficking, we analysed its expression on naïve B cells (28–33). Consistent with increased AAL staining, anti-SLe^X^ staining demonstrated elevated levels of SLe^X^ on patients’ naïve-like CD21^low^ B cells compared to patients’ and HD’s naïve CD21^pos^ B cells. Naïve CD21^pos^ B cells from patients tended to have increased SLe^X^ expression compared to naïve CD21^pos^ B cells from HD, although this did not reach statistical significance (**Figure 2C**). The fucosylation pattern of naïve-like CD21^low^ B cells supports increased α1,3-, α1,4-, and/or α1,6-linked fucose, increased core fucosylation of N-glycans, and increased SLe^X^ epitopes relative to naïve CD21^pos^ B cells.

We further tested whether external factors might influence the glycosylation of B cells in our patients. We did not observe any correlation between age or sex and the glycosylation of CD21^low^ or CD21^pos^ B cells in our patients (**Supplementary Figures S2A, B**). Genetic alterations (chromosome 22 aberrations, *CTLA4* or *KMT2D* mutation) (**Supplementary Figure S2C**) and therapeutic treatments, such as low-dose steroids or sirolimus (**Supplementary Figure S2D**), did not affect the surface glycosylation pattern of naïve-like CD21^low^ and CD21^pos^ B cells. Lastly, we tested if noninfectious complications, which are common in CVID patients with an accumulation of the CD21^low^ B-cell subset, were associated with glycome aberrations. We separated patients according to the presence of splenomegaly, granuloma formation, granulomatous lymphocytic interstitial lung disease (GLILD)/interstitial lung disease (ILD), lymphoproliferation (LP), autoimmune organ manifestation (AIO), autoimmune cytopenia (AIC), enteropathy, and hepatopathy. None of these clinical manifestations was specifically associated with significant changes in the surface glycome (**Supplementary Figure S3**). Overall, these results indicate: (A) that the glycosylation pattern of B cells in CVID is *per se* not strongly influenced by these factors and (B) that the alterations are characteristic for the naïve-like CD21^low^ B-cell subset, in contrast to the CD21^pos^ B-cell compartment.

### 3.2 Cell surface glycome of B-cell subsets from healthy controls

**Figure 3:**
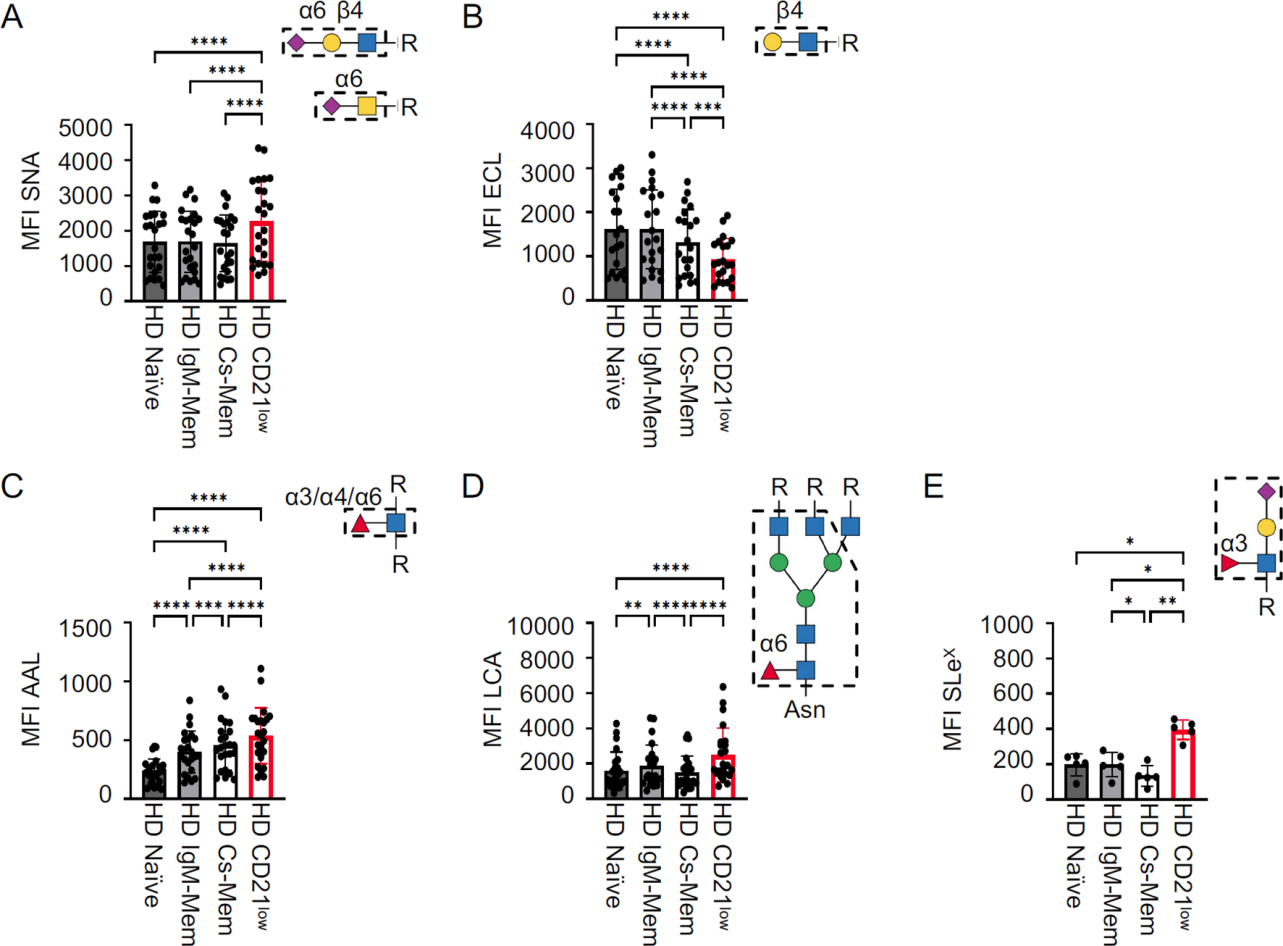
Cell surface glycome of HD B-cell subsets. Statistical analysis of the MFI for **(A)** SNA (*n* = 24), **(B)** ECL (*n* = 21), **(C)** AAL (*n* = 24), **(D)** LCA (*n* = 24), and **(E)** anti-SLe^X^ (*n* = 5) for HD B-cell subpopulations from peripheral blood. CD21^low^ B cells were gated from total B cells, while other subsets were determined from non-CD21^low^ B cells. Lectin/antibody-binding epitopes are illustrated. *p*-values as determined by one-way ANOVA with Tukey’s multiple comparisons test **(A–C, E)** and Friedman’s test with Dunn’s multiple comparisons test **(D)**. *p < 0.05; **p < 0.01; ***p < 0.001; and ****p < 0.0001.

**Figure 4:**
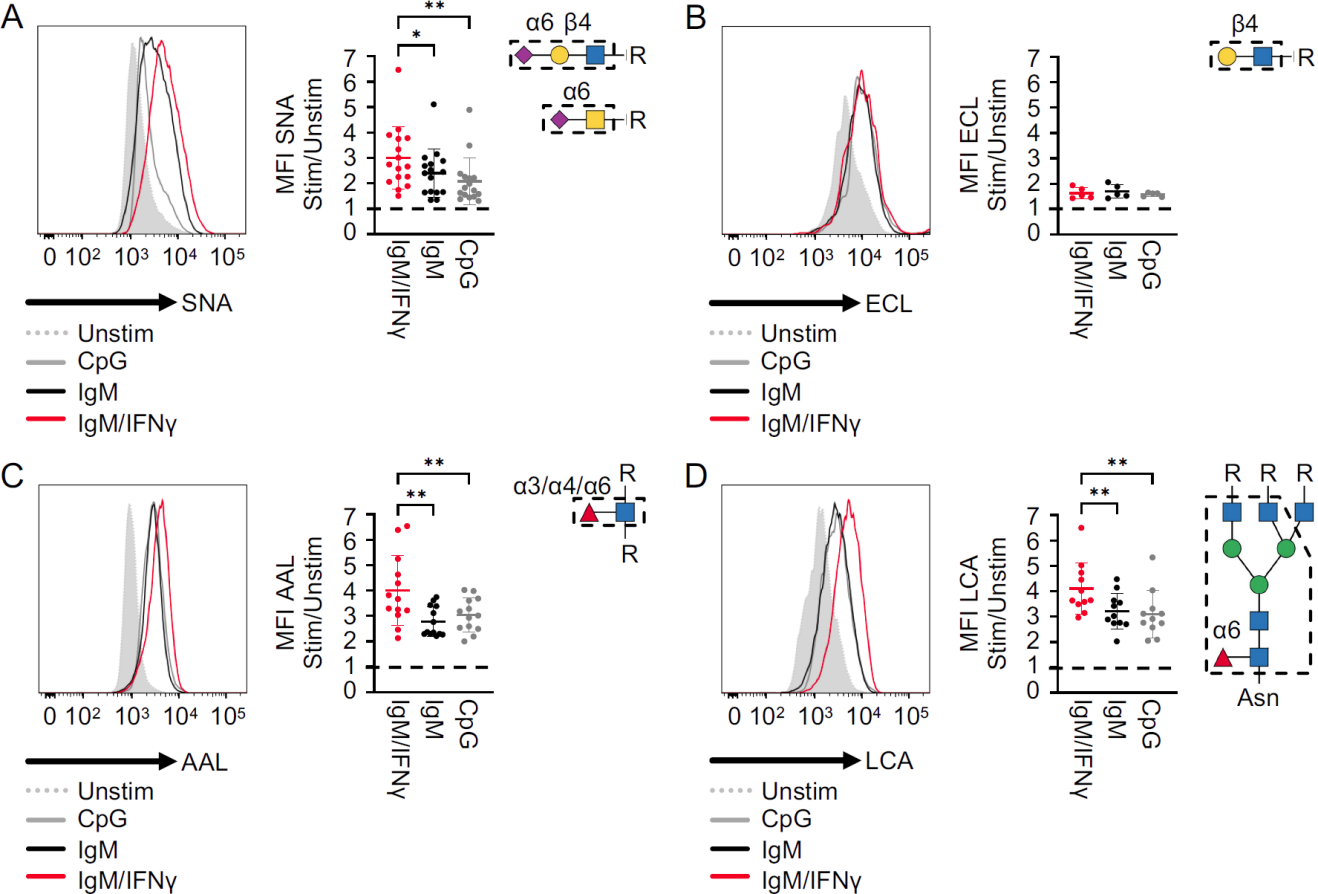
Surface glycome of activated B cells. **(A)** Representative histogram showing the MFI of SNA in activated naïve B cells from a HD after 48 h of *in vitro* stimulation, as indicated, and statistical analysis of the ratios of the MFI of stimulated to unstimulated (*n* = 11). **(B–D)** Results after lectin staining with ECL (*n* = 5), AAL (*n* = 11), and LCA (*n* = 10) as described in **(A)**. Lectin-binding epitopes are illustrated. *p*-values as determined by Friedman’s test with Dunn’s multiple comparisons test **(A, D)** and one-way ANOVA with Tukey’s multiple comparisons test **(B, C)**. *p < 0.05; and **p < 0.01.

Next, to determine whether the altered glycome observed in CD21^low^ B cells from CVID patients is unique to these cells, we compared the glycome of different B-cell subsets derived from peripheral blood and secondary lymphoid organs of HD. Due to the altered composition of CD21^low^ B cells in HD, including classical or atypical memory B-cell subsets and only very low proportions of naïve-like CD21^low^ B cells (34), the analysis in HD refers to “total” CD21^low^ B cells, without differentiating CD27 and IgD expression. Also, in HD, increased SNA and reduced ECL staining corroborated hypersialylation of total CD21^low^ B cells compared to CD21^pos^ naïve, IgM mem, and cs mem B cells (**Figures 3A, B**), indicating that this glycosylation pattern is characteristic of the CD21^low^ B-cell population and not limited to CVID. The degree of α2,6 surface sialylation was similar between the different blood-derived CD21^pos^ B-cell subsets (**Figure 3A**) and, as previously published (16, 35), also between tonsillar B-cell populations (**Supplementary Figures S4A, B**). The level of terminal β1,4-linked galactose was significantly reduced in circulating cs mem B cells compared to naïve and IgM mem B cells, yet still increased compared to CD21^low^ B cells (**Figure 4B**). This difference was not seen for the B-cell populations from secondary lymphoid tissue (**Supplementary Figure S4C**).

CD21^low^ B cells from HD also revealed hyperfucosylation with increased expression of AAL, LCA, and anti-SLe^X^ ligands (**Figures 3C–E**). Unlike the sialylation profile, fucosylation differed between the various CD21^pos^ B-cell populations. We observed increasing levels of AAL ligands from the naïve to the cs mem B-cell compartment, still not reaching the level seen in CD21^low^ B cells (**Figure 3C**), while LCA binding was slightly increased on IgM mem B cells compared to naïve and cs mem B cells (**Figure 3D**). Reduced expression of SLe^X^ was observed in cs mem B cells (**Figure 3E**). Interestingly, in the secondary lymphoid tissue, the strongest AAL and LCA staining was detected in pre-germinal center cells and plasmablasts (**Supplementary Figures S4D, E**). Overall, HD CD21^low^ B cells exhibited the same surface glycosylation pattern as naïve-like CD21^low^ B cells from CVID patients, with the highest levels of α2,6 sialic acids of all investigated B-cell subsets, reduced levels of terminal β1,4 galactose, and increased levels of α1,3, α1,4, and/or α1,6 fucose, N-glycan core fucose, and SLe^X^. The direct comparison of total CD21^low^ B cells in HD and CVID patient-derived naïve-like CD21^low^ B cells did not reveal significant differences (**Supplementary Figure S5**). Minor changes were observed for AAL, which tended to be higher in HD, or LCA, which was slightly lower in HD (**Supplementary Figure S5**). These differences are most likely caused by the changes also observed in the CD21^pos^ memory B-cell subsets compared to naïve, and thus reflect the different composition of the underlying CD21^low^ B-cell subsets in HD and CVID (**Figures 3C, D**). Thus, the altered glycome of CD21^low^ B cells is a general, yet unique and characteristic feature of this B-cell population.

### 3.3 Cell surface glycome after *in vitro* activation of naïve B cells

To investigate the impact of B-cell activation on the glycome, naïve CD21^pos^ B cells from HD were cultured for 48 h *in vitro* with CpG, anti-IgM, anti-IgM/IFN-γ, or without stimulation. These conditions were chosen to distinguish the impact of BCR versus Toll-like receptor signals and, especially, the effect of the combined stimulation with anti-IgM/IFN-γ, which has been shown to provide an important signal for increased expression of the transcription factor T-bet and the differentiation of CD21^low^ B cells (3, 5, 36).

All investigated stimuli increased the expression of SNA ligands on the B-cell surface after 48 h compared to unstimulated cells, but anti-IgM/IFN-γ stimulation induced significantly more α2,6-sialylated epitopes (**Figure 4A**). Unlike our observation of CD21^low^ B cells *ex vivo*, B-cell activation also generally upregulated ECL ligands on the surface of activated cells, indicating a higher overall expression of terminal β1,4-linked galactose, irrespective of increased sialylation (**Figure 4B**). Analysis of fucose residues showed upregulation of AAL and LCA ligands upon each type of activation, reaching the highest levels after anti-IgM/IFN-γ stimulation (**Figures 4C, D**). In conclusion, all investigated conditions of B-cell activation led to the upregulation of SNA, ECL, AAL, and LCA ligands compared to unstimulated cells. A more potent role of anti-IgM/IFN-γ stimulation was visible for the upregulation of SNA, AAL, and LCA ligands, indicating a significantly stronger α2,6 hypersialylation, α1,3, α1,4, and/or α1,6 hyperfucosylation, and N-glycan core fucosylation under this condition.

### 3.4 Glycosylation-related genes in CD21^low^ B cells and anti-IgM/IFN-γ-stimulated B cells

**Figure 5:**
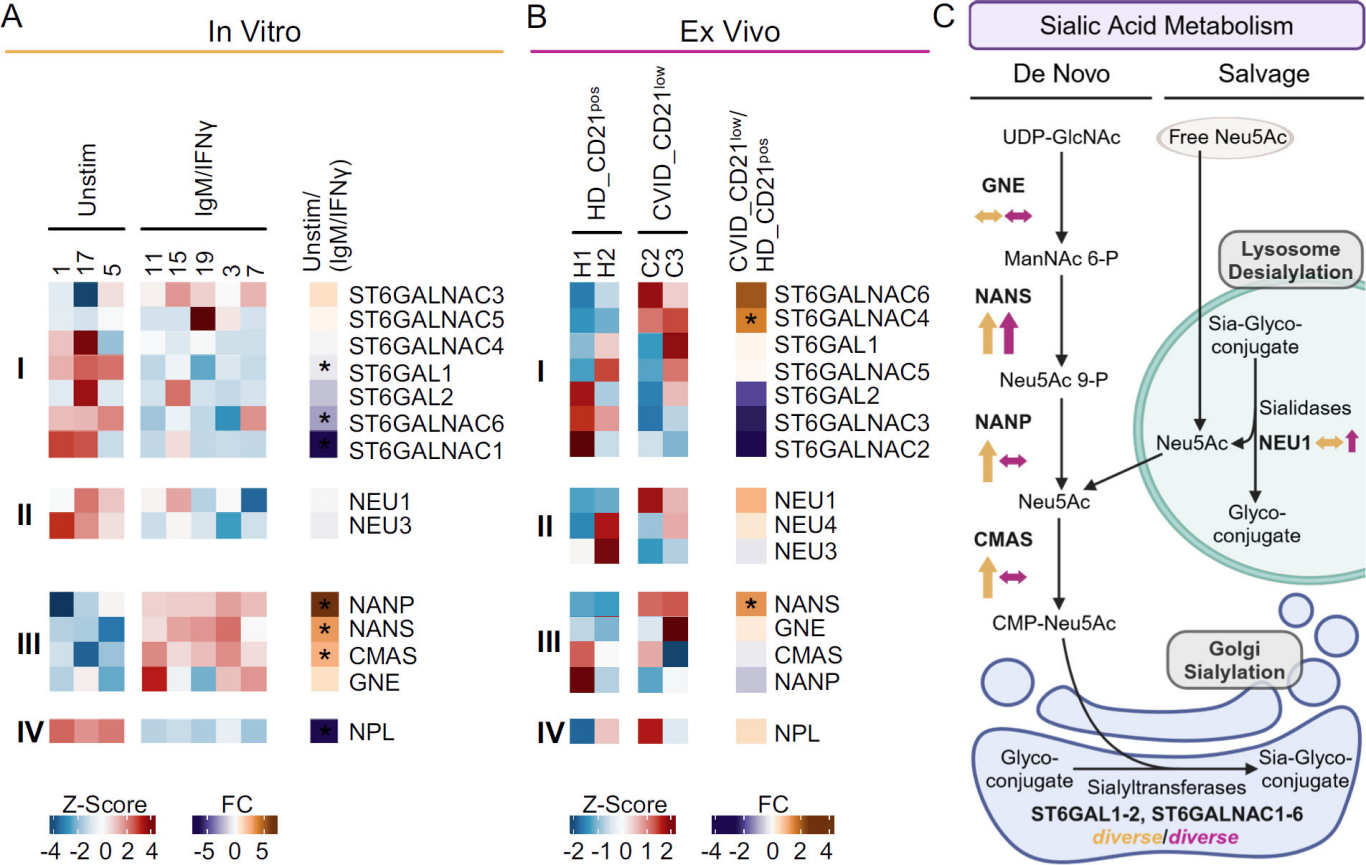
Sialylation-related genes in anti-IgM/IFN-γ-activated B cells and naïve-like CD21^low^ B cells. **(A)** Heatmap of selected genes regulating sialylation, as determined by RNAseq of activated naïve B cells after *in vitro* stimulation with anti-IgM/IFN-γ compared to unstimulated cells. The expression of genes encoding for α2,6 sialyltransferases (i), sialidase (ii), enzymes involved in CMP-sialic acid *de novo* synthesis (iii), and sialic acid degradation (iv) is depicted. **(B)** Heatmap of sialylation-regulating genes identified by RNAseq of naïve-like CD21^low^ B cells from CVID patients compared to naïve CD21^pos^ B cells from HD *ex vivo*. **(C)** Schematic overview of gene expression involved in sialic acid metabolism after anti-IgM/IFN-γ *in vitro* stimulation (yellow) and in naïve-like CD21^low^ B cells *ex vivo* (purple). Pathways include sialic acid salvage and CMP-Neu5Ac *de novo* synthesis as substrates for sialyltransferases (42, 43). Increased (↑), decreased (↓), or not clearly deviating (↔) gene expression is indicated. Large arrows indicate significant changes, while small ones indicate clear trends without reaching significance. The correct illustration of *CMAS* in the nucleus (77) was omitted for better visualisation. UDP, uridine diphosphate; GlcNAc, N-acetyl glucosamine; ManNAc, N-acetyl mannosamine; P, phosphate; Neu5Ac, N-acetyl neuraminic acid; CMP, cytidine monophosphate. **(C)** was created with BioRender.com. *p < 0.05.

**Figure 6:**
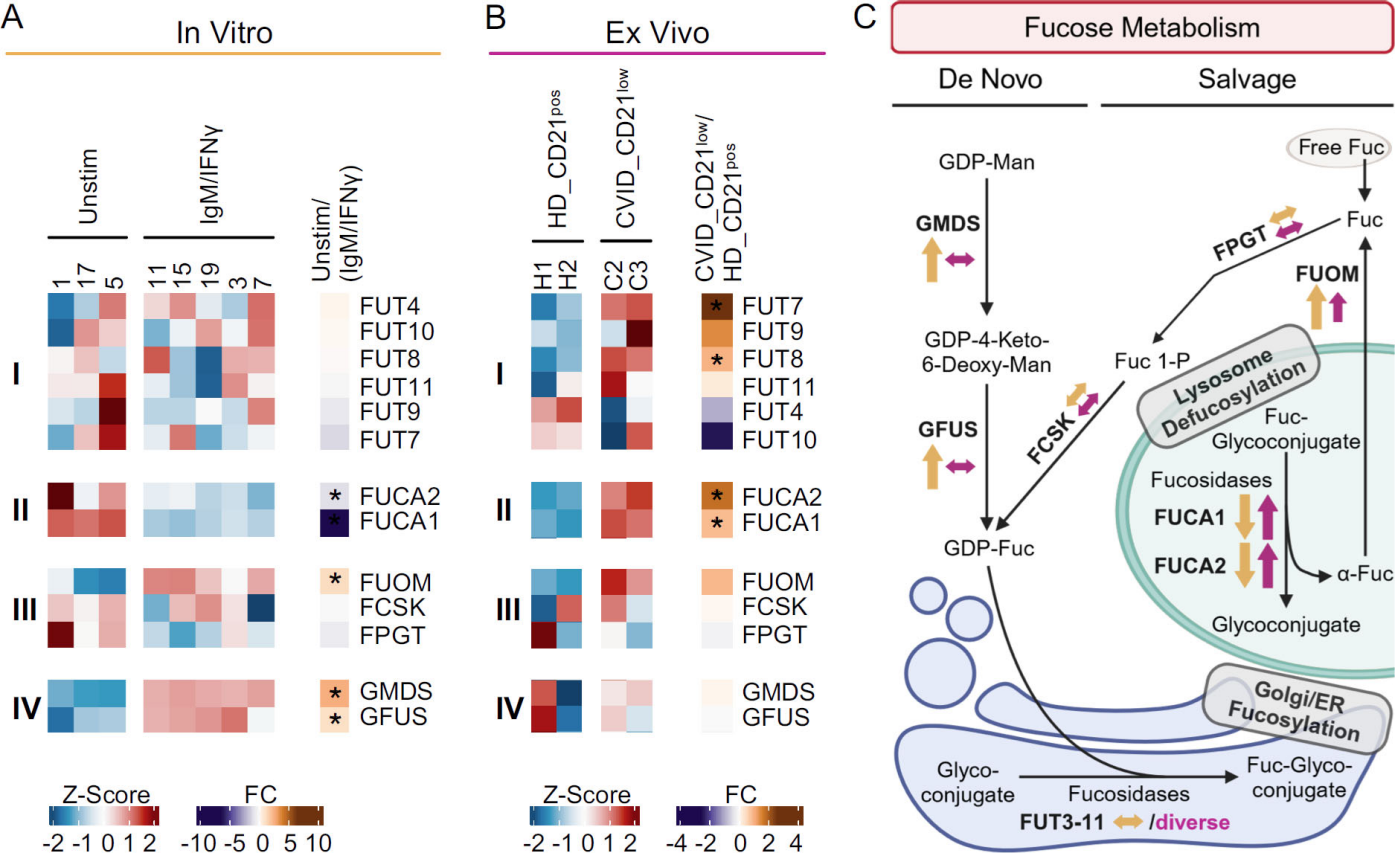
Fucosylation-related genes in anti-IgM/IFN-γ-activated B cells and naïve-like CD21^low^ B cells. **(A)** Heatmap of selected genes of fucosylation in 48 h anti-IgM/IFN-γ-stimulated naïve B cells (IgM/IFN-γ) compared to unstimulated cells. The expression of genes encoding for α1,3, α1,4, α1,6 and fucosyltransferases (i), fucosidases (ii), enzymes of the GDP-fucose salvage (iii), and *de novo* synthesis (iv) is depicted. **(B)** Heatmap of genes in naïve-like CD21^low^ B cells from CVID patients compared to naïve CD21^pos^ B cells from HD, as described in **(A)**. **(C)** Fucose salvage and GDP-fucose *de novo* synthesis as substrates for fucosyltransferases (48, 49). Increased (↑), decreased (↓), or not clearly deviating (↔) gene expression is indicated for anti-IgM/IFN-γ-stimulated B cells (yellow) and naïve-like CD21^low^ B cells (purple). Large arrows indicate significance, while small ones a clear trend without reaching significance. The correct illustration of the extracellular localisation of *FUCA2* (78) was omitted for better visualization. GDP, guanosine diphosphate; Man, mannose; Fuc, fucose; ER, endoplasmic reticulum. **(C)** was created with BioRender.com. *p < 0.05.

The transcription of glycosylation-related genes is a key factor in the cellular regulation of expressed glycans (37). Given the prominent role of anti-IgM/IFN-γ stimulation in the differentiation of the naïve-like CD21^low^ B-cell phenotype (5), we compared DEGs from anti-IgM/IFN-γ-activated and unstimulated B cells from HD to DEGs of naïve-like CD21^low^ B cells from CVID patients and naïve CD21^pos^ B cells from HD *ex vivo*. A comparative analysis of selected genes involved in sialylation (**Figure 5**) and fucosylation (**Figure 6**) was performed. We focused on the expression of genes encoding for α2,6 sialyltransferases and α1,3, α1,4, or α1,6 fucosyltransferases, which are responsible for the generation of SNA, AAL, or LCA ligands, as well as genes contributing to the synthesis of the nucleotide sugars cytidine monophosphate (CMP)-sialic acid and guanosine diphosphate (GDP)-fucose as substrate for the respective glycosyltransferases.

Anti-IgM/IFN-γ *in vitro*-stimulated B cells revealed diverse deviations in the expression of sialyltransferases that generate SNA epitopes by linking α2,6 sialic acid to galactose or N-acetyl galactosamine (38, 39). Only *ST6GALNAC3* tended to be upregulated in the activated B cells, while *ST6GALNAC4* and *ST6GALNAC5* were not differentially expressed, *ST6GAL2* tended to be downregulated, and *ST6GAL1*, *ST6GALNAC1*, and *ST6GALNAC6* were significantly downregulated (**Figure 5Ai**). Genes for sialidases (also known as neuraminidases) that remove sialic acid from sialic acid-containing glycans (40, 41), such as *NEU1* and *NEU3*, were not differentially expressed *in vitro* (**Figure 5Aii**). Genes regulating the *de novo* pathway of CMP-Neu5Ac synthesis (*NANS*, *NANP*, and *CMAS* but not *GNE*) were significantly upregulated in anti-IgM/IFN-γ-activated B cells (**Figure 5Aiii**) (42, 43). *NPL*, involved in the degradation of sialic acid, was significantly downregulated (**Figure 5Aiv**) (42, 44).

The analysis of differentially expressed genes in naïve-like CD21^low^ B cells compared to naïve HD B cells *ex vivo* revealed a significant upregulation of *ST6GALNAC4* and increased *ST6GALNAC6* expression, though not reaching significance. The expression of other α2,6 sialyltransferases, such as *ST6GAL1*, *ST6GAL2*, *ST6GALNAC2*, *ST6GALNAC3*, and *ST6GALNAC5* were unremarkable or tended to be downregulated (**Figure 5Bi**). The sialidase *NEU1* tended to be upregulated in naïve-like CD21^low^ B cells, while others, such as *NEU3* and *NEU4*, were not differentially expressed (**Figure 5Bii**). In contrast to the *in vitro*- activated B cells, *NANS* was the only significantly upregulated gene related to the *de novo* synthesis of CMP-sialic acid in naïve-like CD21^low^ B cells *ex vivo* (42, 43), while *GNE*, *NANP*, and *CMAS* were not differentially expressed (**Figure 5Biii**). *NPL* was not differentially expressed *ex vivo* (**Figure 5Biv**).

Overall, the results suggest an increased *de novo* synthesis of CMP-sialic acid in anti-IgM/IFN-γ-activated B cells and reduced sialic acid degradation potentially leading to increased substrate for sialyltransferases as the basis for α2,6 hypersialylation. In contrast, the α2,6 hypersialylation in naïve-like CD21^low^ B cells may reflect a multifactorial basis involving the *de novo* and salvage synthesis of CMP-sialic acid and upregulation of individual sialyltransferases (**Figure 5C**).

None of the fucosyltransferases *FUT3* to *FUT11*, linking fucose α1,3, α1,4, and/or α1,6 to N-acetyl glucosamine (45–47) and thus elicit AAL or LCA ligands, were differentially expressed in anti-IgM/IFN-γ-stimulated B cells (**Figure 6Ai**). In contrast, the *FUCA1* and *FUCA2* which remove fucose from fucosylated glycans, were significantly downregulated (**Figure 6Aii**) (40, 41). While activated B cells significantly upregulated *FUOM*, further genes of the fucose salvage pathway (*FPGT, FCSK*) were not differentially expressed (**Figure 6Aiii**) (48, 49). However, with *GMDS* and *GFUS*, all genes involved in the *de novo* synthesis of GDP-fucose were significantly upregulated *in vitro* (**Figure 6Aiv**) (48, 49).

Our analysis of naïve-like CD21^low^ B cells *ex vivo* exhibited diverse expression of fucosyltransferases. *FUT7*, one of the α1,3 fucosyltransferases with the highest potency in creating the SLe^X^ epitope (47, 50), and *FUT8*, the only known fucosyltransferase that can link fucose α1,6 to form N-glycan core fucose (45), were significantly upregulated (**Figure 6Bi**). The significant upregulation of both fucosidases, *FUCA1* and *FUCA2*, in naïve-like CD21^low^ B cells (**Figure 6Bii**) and the trend toward upregulation of *FUOM* suggests increased recycling of fucose in naïve-like CD21^low^ B cells, in the presence of regular expression of the other genes involved in the fucose salvage pathway, *FPGT* and *FCSK* (**Figure 6Biii**). Genes of the *de novo* synthesis of GDP-fucose were not differentially expressed (**Figure 6Biv**).

As seen for sialylation, *in vitro* anti-IgM/IFN-γ-activated B cells upregulate genes involved in the *de novo* synthesis of GDP-fucose, potentially resulting in increased substrate for fucosyltransferases as basis for hyperfucosylation. In contrast, the basis in naïve-like CD21^low^ B cells *ex vivo* seems to involve the upregulation of individual genes of fucosyltransferases and the fucose salvage pathway (**Figure 6C**).

## 4 Discussion

During lymphocyte development, the cellular glycome undergoes continuous changes that are believed to be relevant for differentiation, cell–cell interaction, and activation (51, 52). Although the understanding of the highly complex and dynamic glycome has advanced in recent years, the influence of specific glycan structures on individual cell types and differentiation phases, as well as their significance to immune system integrity and dysfunction, remains incomplete. Translation is further complicated by differences between humans and mice, despite conserved processes (53). In this study, we report the unique and characteristic glycosylation pattern of CD21^low^ B cells.

Our data imply that naïve-like CD21^low^ B cells from CVID patients and CD21^low^ B cells from healthy controls exhibit a comprehensively restructured cell surface glycome, characterised by uniquely high levels of α2,6-linked sialic acid and fucose. The common upregulation of α2,6 sialyltransferases (*ST6GALNAC4*, *ST6GALNAC6)*, along with *NEU1* and *NANS* in naïve-like CD21^low^ B cells from CVID patients, potentially increases substrate availability through enhanced sialic acid recycling and/or *de novo* synthesis of CMP-sialic acid (42, 43), suggesting a multifactorial basis for α2,6 hypersialylation in these cells. A multifactorial genesis likely underlies the hyperfucosylation observed in CD21^low^ B cells, as the upregulation of *FUT7*, *FUT8*, *FUCA1*, *FUCA2*, and *FUOM* in naïve-like CD21^low^ B cells from CVID patients suggests the relevance of fucosyltransferases and their substrate availability, potentially stemming from increased recycling of fucose (48, 49). While *FUT8* encodes a fucosyltransferase uniquely responsible in linking fucose α1,6, thereby forming N-glycan core fucose (45), the fucosyltransferase encoded by *FUT7* exhibits the highest potency in creating the α1,3 fucose-containing selectin ligand SLe^X^ (50). The upregulation of these two fucosyltransferases in patients’ CD21^low^ B cells may consequently contribute to the observed hyperfucosylation in these cells. Although the glycosidases *NEU1*, *FUCA1*, and *FUCA2*, which remove sialic acid and fucose from glycans (40, 41), are upregulated CD21^low^ B cells, we observed increased α2,6 sialylation and α1,3, α1,4 and/or α1,6 fucosylation on CD21^low^ B cells, suggesting a potentially ongoing reconstruction of the surface glycome. This hypothesis is strengthened by the increased activity of *NEU1*, which hydrolyses α2,3-linked sialic acid more rapidly than α2,6- or α2,8-linked sialic acid (40, 54), and by *FUCA1* and *FUCA2*, which preferentially hydrolyse fucose α1,2-linked to galactose than fucose α1,3-, α1,4-, and α1,6-linked to N-acetyl glucosamine (41).

Interestingly, the CD21^low^ B-cell-related CD11c^high^ B-cell population in SLE (55, 56) exhibits significant transcriptomic similarities in the expression of glycosylation-related genes, with marked upregulation of *ST6GALNAC4*, *ST6GALNAC6*, *NEU1*, *NANS*, *FUT8*, *FUCA1*, *FUCA2*, and *FUOM* (57). This further supports our hypothesis that hypersialylation and hyperfucosylation are general features of the CD21^low^ B-cell population. In contrast, hypofucosylation has been reported in SLE-derived B cells without specific changes in the double-negative (CD27^neg^/IgD^neg^) atypical memory B-cell population (16). This finding clearly contrasts with the hyperfucosylation observed in naïve-like CD21^low^ B cells in our study. Whether this represents a true difference between naïve-like CD21^low^ B cells and double-negative (CD27^neg^/IgD^neg^) atypical memory B cells or a specific finding related to altered glycosylation in SLE requires further investigation.

Anti-IgM/IFN-γ stimulation provides a nonredundant signal during the differentiation of CD21^low^ B cells and induces the initial changes toward the CD21^low^ B-cell phenotype (5). Consistent with our hypothesis of increased sialylation and fucosylation on CD21^low^ B cells *ex vivo*, the combined anti-IgM/IFN-γ stimulation was also the strongest inducer of α2,6 hypersialylation and α1,3, α1,4, and/or α1,6 hyperfucosylation in naïve B cells among all the investigated stimuli. Significant upregulation of *NANS*, *NANP*, *CMAS*, *GMDS*, and *GFUS*—the majority of genes involved in the *de novo* synthesis of CMP-sialic acid and GDP-fucose—suggests a central role for these pathways *in vitro*, potentially providing increased substrate for the respective sialyl- and fucosyltransferases (42, 43, 48, 49). Thus, anti-IgM/IFN-γ-driven B-cell activation most likely contributes to the altered glycome during the differentiation of CD21^low^ B cells. Although presenting a similar surface glycosylation pattern, the mechanisms differ from those observed in naïve-like CD21^low^ B cells *ex vivo*, suggesting potential differences in glycosylation during the differentiation and maintenance of these cells. Still, we cannot exclude the effects of limiting culture conditions on the availability of respective substrates.

The understanding of these aberrations in the glycome of normal human B cells remains ambiguous. In mice, increased sialylation limits the antigen-presenting potential (58–60). Sialic acids on antigen-presenting cells or the antigen itself can even induce B-cell tolerance, depending on interactions with Siglecs on the interacting cell, such as CD22 (Siglec 2) and/or Siglec G, the murine orthologue of Siglec 10 (61–64). Thus, in the context of increased frequency of autoreactive BCR specificities in the CD21^low^ B-cell population (7, 8, 65, 66), hypersialylation may reflect an attempt to regulate an exacerbated immune response in autoimmune settings. On the other hand, reduced levels of terminal β1,4-linked galactose on CD21^low^ B cells may actively contribute to immune dysregulation by limiting the secretion of highly expressed galectin 1 from naïve-like CD21^low^ B cells, potentially favouring its intracellular retention (67). Since secreted galectin 1 counteracts Th1-driven immune responses (68, 69), reduced galectin 1 release by this B-cell subset may itself support a Th1 bias in its immediate neighbourhood, potentially contributing to a preferential type 1 immune response in different CD21^low^ B-cell-associated disease conditions (3, 5, 70, 71). Moreover, upregulated SLe^X^ on CD21^low^ B cells may support their accumulation in inflamed tissues, such as the synovia of inflamed joints in rheumatoid arthritis, where the expression of SLe^X^, recognising endothelial selectins, is increased (28–33). Specific glycans mediate distinct functions. One of the most prominent examples is IgG: α2,6-linked sialylation of IgG conveys anti-inflammatory properties and reduces antibody-dependent cell-mediated cytotoxicity (ADCC) (72). Similarly, hyperfucosylation decreases ADCC (73). Notably, the secretion of IgG is low in antibody-deficient CVID patients. We cannot exclude the possibility that Ig substitution-mediated processes also play a role in the glycome changes observed in CD21^low^ B cells from CVID patients, but given the strong similarities to the glycome of CD21^low^ B cells in HD, this does not seem to be crucial. Unfortunately, the determination of the sialylation or fucosylation status of distinct surface proteins of CD21^low^ B cells, such as CD45 or the BCR, known to be strongly influenced by glycosylation, was not feasible due to the limited number of primary B cells from immunodeficient patients.

It is noteworthy that increased sialylation, enhanced N-glycan core fucosylation, and elevated levels of SLe^X^ on CD21^low^ B cells are also seen in tumour cells, altering their cell interactions and modulating signal transduction (74). Tumour cells benefit from these aberrations, which foster their migration, promote their survival, and aid in immune evasion. These insights have opened new therapeutic strategies (75). The direct transfer of, for example, sialidase-conjugated antibodies mediating targeted desialylation of mammary carcinoma cells promoted the infiltration and activation of immune cells (76). A deeper understanding of the function and consequences of specific alterations in the glycome in human B cells is required before therapies targeting sialylation or fucosylation in CD21^low^ B cells, or directly modulating glycan–lectin interactions, can be applied as a therapeutic approach in CVID.

In summary, naïve-like CD21^low^ B cells from CVID patients and CD21^low^ B cells from healthy controls exhibit a unique glycosylation pattern, expressing the highest levels of α2,6 sialic acid and high levels of α1,3-, α1,4-, and/or α1,6-linked fucose, including N-glycan core fucose and of SLeX. Unlike the more dynamic profile of fucosylation in different B-cell subpopulations, hypersialylation was unique for the CD21^low^ B-cell phenotype. *In vitro* experiments indicate that these alterations are especially induced by the costimulation with anti-IgM/IFN-γ, which represents a key mechanism in the differentiation of the T-bet-expressing CD21^low^ B-cell population. RNAseq data depict a fundamental reorganisation of the cellular glycosylation machinery during and after the differentiation of this unique B-cell subset. Our findings add a new aspect to the functional consequences of a chronic type I immune response in complicated CVID patients, which may become relevant if glycome-targeting strategies are incorporated into the therapeutic arsenal against autoimmune diseases.

## Data Availability

The datasets for anti-IgM/IFN-γ *in vitro*-stimulated B cells compared to unstimulated cells, and for naïve-like CD21^low^ B cells *ex vivo* compared to HD CD21^pos^ B cells, are available in the Gene Expression Omnibus data repository of the National Center for Biotechnology Information (21) under accession numbers GSE181739 and GSE148163, respectively.

## Glossary

AAL: *Aleuria aurantia* lectin
ADCC: antibody-dependent cell-mediated cytotoxicity
AIC: autoimmune cytopenia
AIO: autoimmune organ manifestation
APC: allophycocyanin
BCR: B-cell receptor
CMAS: cytidine monophosphate N-acetylneuraminic acid synthetase
Cs mem: class-switched memory
CMP: cytidine 5′-monophosphate
CVID: common variable immunodeficiency
DEGs: differentially expressed genes
ECL: *Erythrina cristagalli* lectin
FCSK: fucose kinase
FPGT: fucose-1-phosphate guanylyltransferase
FUCA1: alpha-L-fucosidase 1
FUCA2: alpha-L-fucosidase 2
FUT3-11: fucosyltransferase 3-11
FUOM: fucose mutarotase
GDP: guanosine 5′-diphosphate
GFUS: GDP-L-fucose synthase
GLILD: granulomatous lymphocytic interstitial lung disease
GMDS: GDP-mannose 4,6-dehydratase
GNE: glucosamine (UDP-N-acetyl)-2-epimerase/N-acetylmannosamine kinase
HD: healthy donor
IFN-γ: interferon γ
IgM mem: IgM memory
ILD: interstitial lung disease
IMDM: Iscove’s modified Dulbecco’s medium
LCA: *Lens culinaris* agglutinin
LP: lymphoproliferation
MFI: mean fluorescence intensity
NANP: N-acetylneuraminic acid phosphatase
NANS: N-acetylneuraminate synthase
NEU1: neuraminidase 1
NEU3: neuraminidase 3
NEU4: neuraminidase 4
NPL: N-acetylneuraminate pyruvate lyase
PBMCs: peripheral blood mononuclear cells
RA: rheumatoid arthritis
RNAseq: RNA-sequencing
SD: standard deviation
SLe^X^: sialyl-Lewis X
SNA: *Sambucus nigra* agglutinin
SLE: systemic lupus erythematosus
ST6GAL1-2: ST6 Beta-galactoside alpha-2,6-sialyltransferase 1-2
ST6GALNAC1-6: ST6 N-acetylgalactosaminide alpha-2,6-sialyltransferase 1-6
T-bet: T-box expressed in T cells.
