# Supplementary material for: CD21low B cells reveal a unique glycosylation pattern with hypersialylation and hyperfucosylation
Source: Front Immunol. 2025 Feb 12;16:1512279. doi: 10.3389/fimmu.2025.1512279 (PMC11861550; doi:10.3389/fimmu.2025.1512279)
Supplement: Supplementary file 1 [file DataSheet1.docx]

Supplementary Material

# Supplementary Table

Supplementary Table 1. Patient cohort

| **No** | **Sex** | **Age** | **Genetics** | **CVID** | **Granu-loma** | **GLILD/ ILD** | **LP** | **Spleno** | **AIO** | **AIC** | **Entero-pathy** | **Hepato-pathy** | **IS** |
| --- | --- | --- | --- | --- | --- | --- | --- | --- | --- | --- | --- | --- | --- |
| **1** | f | 37 | - | c | - | Yes | Yes | Yes | - | - | - | - | Steroids |
| **2** | m | 57 | - | c | - | - | - | Yes | - | - | - | - | - |
| **3** | m | 40 | - | c | - | - | Yes | Yes | - | - | - | - | - |
| **4** | f | 33 | Chr22 del | c | - | - | - | Yes | - | Yes | - | - | - |
| **5** | f | 46 | - | c | - | - | Yes | Yes | - | Yes | - | - | - |
| **6** | m | 49 | - | c | Yes | Yes | Yes | Yes | - | - | - | - | Steroids, Abatacept |
| **7** | f | 61 | Chr22q11 | c | - | Yes | - | - | Yes | Yes | - | - | Steroids |
| **8** | f | 28 | - | c | - | Yes | Yes | - | - | Yes | - | Yes | Sirolimus |
| **9** | m | 60 | - | c | Yes | - | Yes | - | - | Yes | - | - | Steroids |
| **10** | m | 55 | - | c | - | - | Yes | Yes | - | - | Yes | - | - |
| **11** | m | 74 | - | c | - | - | - | Yes | Yes | - | - | - | - |
| **12** | f | 25 | - | c | - | Yes | Yes | Yes | - | Yes | - | - | - |
| **13** | f | 58 | - | c | Yes | Yes | Yes | Yes | Yes | - | Yes | Yes | Steroids |
| **14** | f | 52 | - | c | - | - | Yes | Yes | - | - | Yes | Yes | - |
| **15** | f | 59 | *CTLA4* | c | Yes | Yes | Yes | Yes | - | - | Yes | - | Steroids |
| **16** | f | 71 | - | c | - | Yes | - | Yes | - | - | - | - | - |
| **17** | m | 62 | - | c | - | Yes | Yes | Yes | - | - | - | Yes | Steroids |
| **18** | f | 38 | Kabuki (*KMT2D*) | c | - | Yes | - | Yes | - | Yes | - | - | - |
| **19** | m | 43 | *CTLA4* | c | - | Yes | - | Yes | - | - | Yes | - | - |
| **20** | f | 55 | - | c | - | - | - | - | - | Yes | - | Yes | - |
| **21** | f | 62 | - | c | Yes | Yes | Yes | Yes | Yes | Yes | - | Yes | Abatacept |
| **22** | m | 72 | - | c | Yes | Yes | Yes | Yes | - | - | - | Yes | Steroids |
| **23** | f | 62 | - | c | - | - | - | Yes | - | - | - | - | - |

Supplementary Table 1. Patient cohort. Epidemiological and clinical information of patients examined in this study. CVID common variable immunodeficiency, GLILD Granulomatous lymphocytic interstitial lung disease, ILD Interstitial lung disease, LP Lymphoproliferation, Spleno Splenomegaly, AIO Autoimmune organ manifestation, AIC Autoimmune cytopenia. IS Immunosuppression. All patients received Immunoglobulin substitution. Patients under steroid therapy were treated with low dose steroids.

# Supplementary Figures


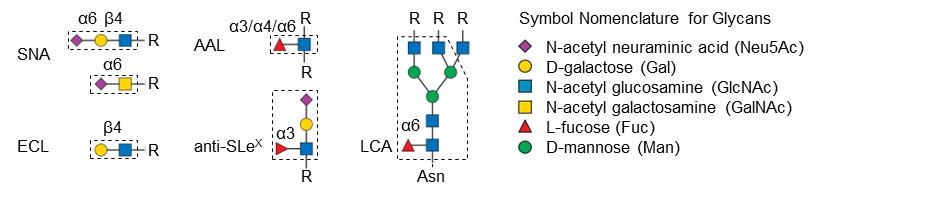


Supplementary Figure 1. Glycan binding epitopes of lectins and antibodies

SNA preferentially binds N-acetyl neuraminic acid, the sialic acid predominantly found in humans (1), α2,6-linked to galactose or N-acetyl galactosamine or 6-O-sulfated N-acetyl lactosamine. ECL binds terminal galactose β1,4-linked to N-acetyl glucosamine. AAL detects fucose α1,3-, α1,4- and α1,6-linked to N-acetyl glucosamine while LCA recognizes α1,6-fucosylated N-glycans. The glycan epitope detected by the anti-Sialyl Lewis X antibody is shown. Symbols representing respective monosaccharides according to the Symbol Nomenclature for Graphical Representation of Glycans are shown. Glycans were created using the web software DrawGlycan (2).


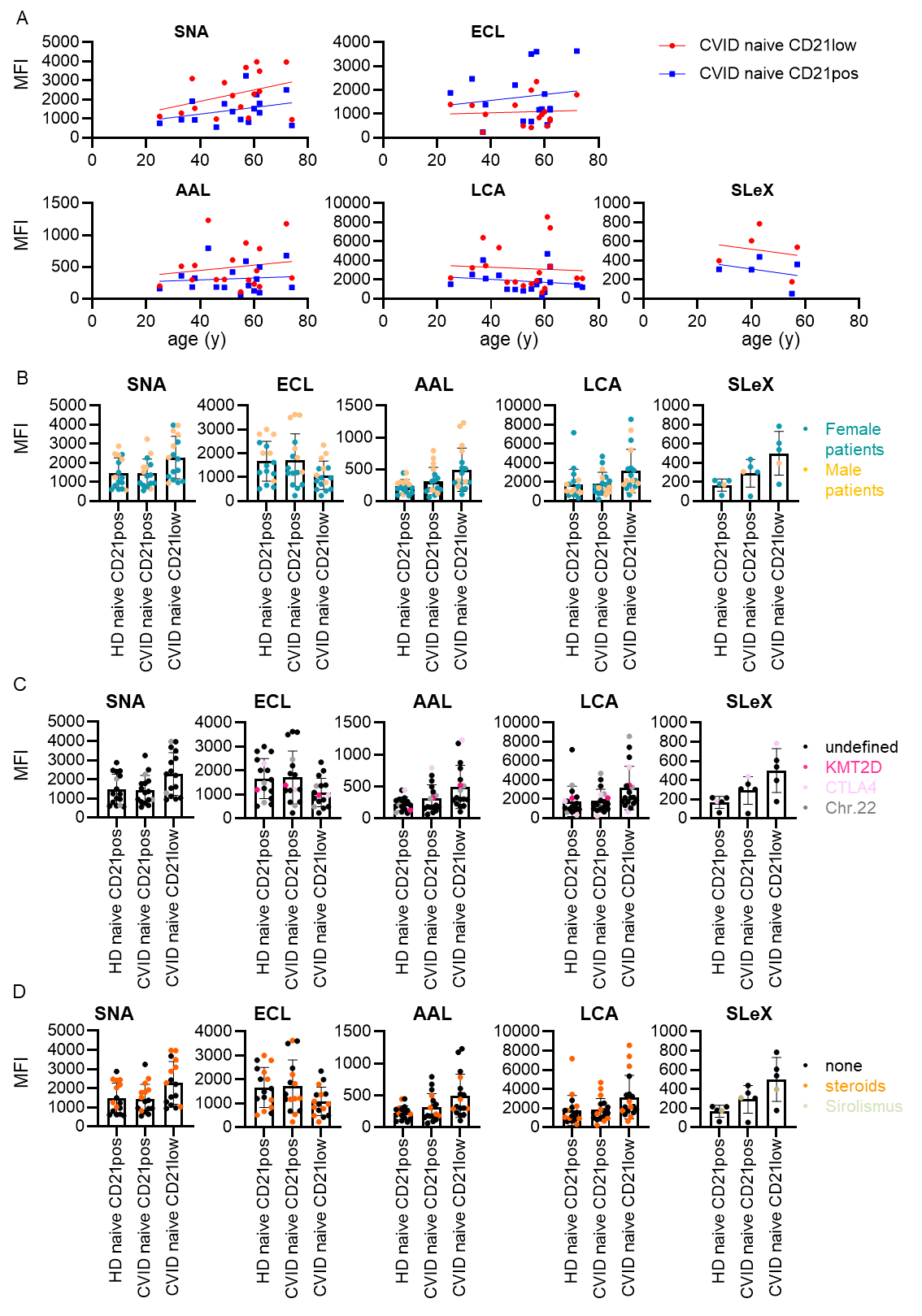
Supplementary Figure 2. Association of glycosylation and external determinants

**(A)** Correlation of the MFI of SNA, ECL, AAL, LCA and anti-SLe^X^ and the age of CVID patients. Naïve-like CD21^low^ B cells are depicted in red, CD21^pos^ B cells in blue. **(B)** Analysis of the MFI of SNA, ECL, AAL, LCA and anti-SLe^X^ in naïve CD21^pos^ B cells from HD, and naïve CD21^pos^ and naïve-like CD21^low^ B cells from CVID patients. Male patients (and the respective day controls) are depicted in yellow, female patients and controls in turquois. **(C)** Analysis as shown in (B) differentiating patients with defined genetic defects. The *KMT2D* mutated patient and the respective day control are shown in pink, *CTLA4* in rose, and patients with Chromosome 22 alterations are labelled in grey. **(D)** Analysis as shown in (B) and (C) showing therapeutic treatments. All patients received Ig substitution. Low dose steroids are depicted in orange, one patient under Sirolimus treatment is shown in light green.


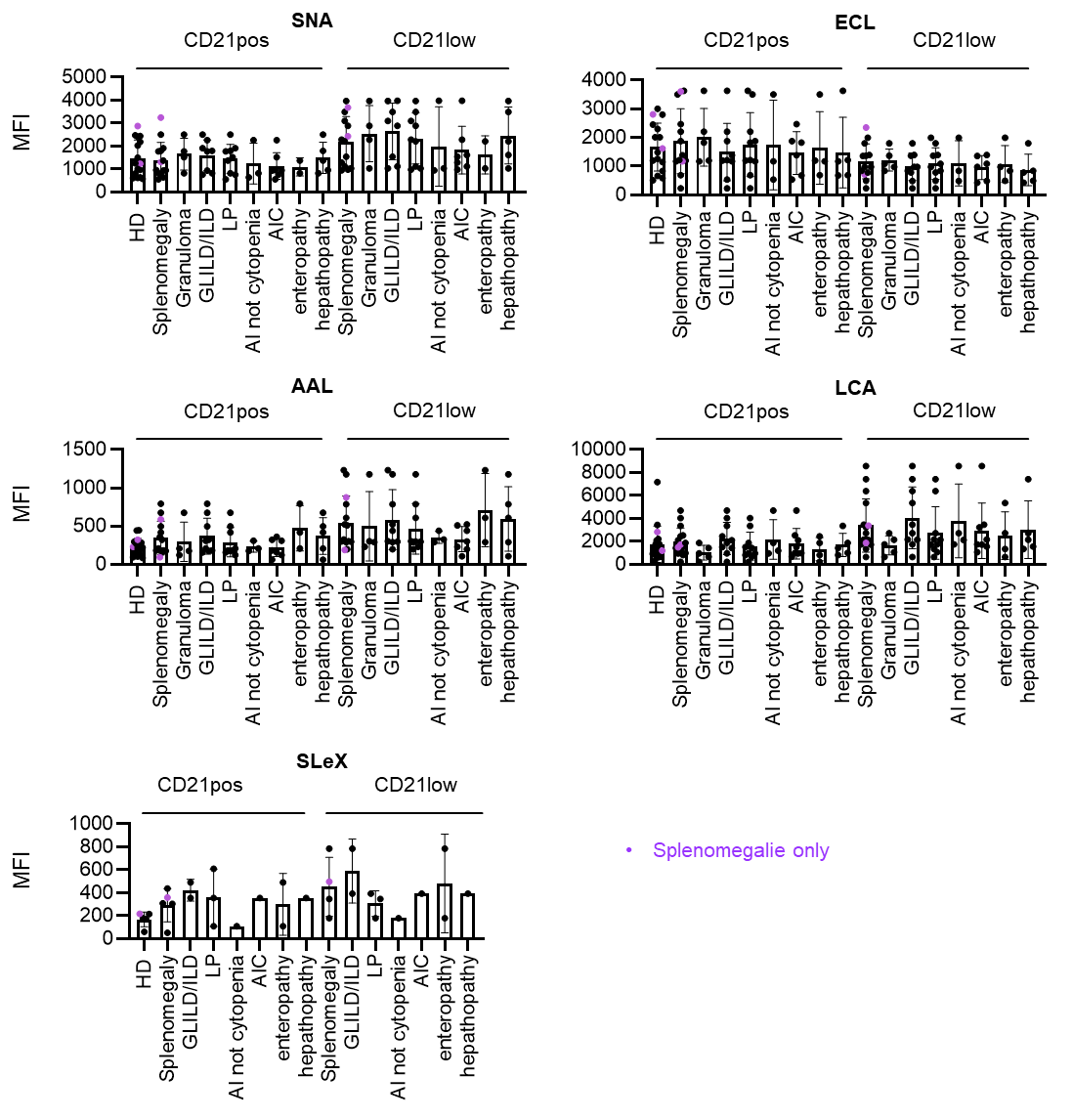


Supplementary Figure 3. Association of glycosylation and clinical manifestations

Analysis of the MFI of SNA, ECL, AAL, LCA and anti-SLe^X^ in naïve CD21^pos^ B cells from HD, and naïve CD21^pos^ and naïve-like CD21^low^ B cells from CVID patients. Data were separated according to additional non-infectious complications of the patients. Patients with splenomegaly only, and the respective day controls are labelled in violet. Patients with multiple complications appear repeatedly in the table.


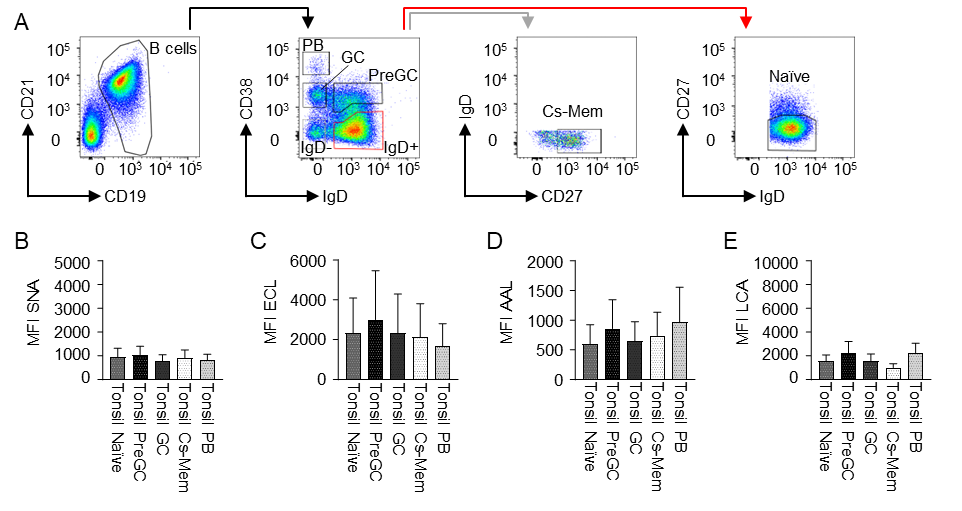


Supplementary Figure 4. Surface glycome of tonsillar B-cell subpopulations ex vivo

(A) Gating for tonsillar B-cell subsets from HD ex vivo. Pre-germinal center cells (preGC), germinal center cells (GC), plasmablasts (PB), tonsillar class-switched memory B cells (Cs-Mem) and tonsillar naïve B cells were gated from CD19^pos^CD21^pos-low^ B cells. (B-E) Bar plots show the MFI of SNA (n=4), ECL (n=4), AAL (n=4) and LCA (n=4) on tonsillar B-cell subsets as described. Statistical analysis was performed using one-way ANOVA with Tukey’s multiple comparisons test (B-E).


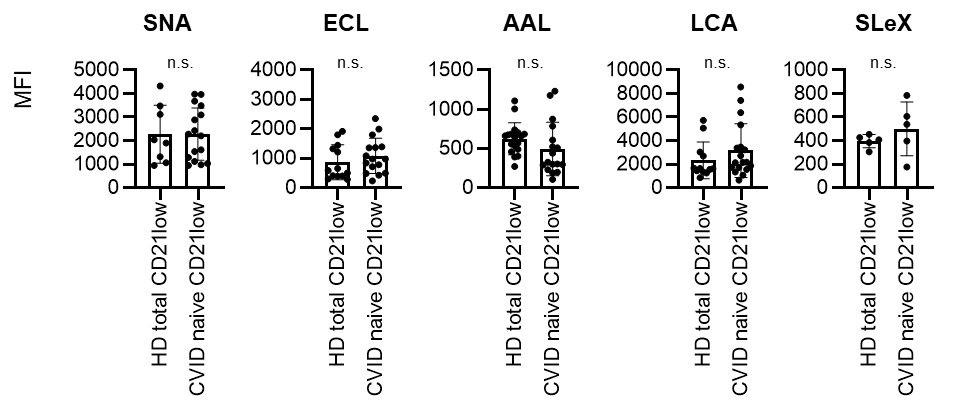


Supplementary Figure 5. Comparison of CD21^low^ B cells in health and disease

SNA, ECL, AAL, LCA and anti-SLe^X^ in total CD21^low^ B cells from HD and in naïve-like CD21^low^ B cells from CVID patients. Statistical analysis was performed using unpaired t tests (SNA), paired t test (SLe^X^) or Mann-Whitney tests (ECL, AAL, LCA). N.s. not significant.

References

1. Varki A, Cummings RD, Esko JD, Stanley P, Hart GW, Aebi M et al., editors. Essentials of Glycobiology. 3rd ed. Cold Spring Harbor, New York: Cold Spring Harbor Laboratory Press; 2017.

2. Cheng K, Zhou Y, Neelamegham S. DrawGlycan-SNFG: a robust tool to render glycans and glycopeptides with fragmentation information. Glycobiology 2017; 27(3):200–5.
